# A case–control study evaluating the unnecessary use of intravenous broad-spectrum antibiotics in presumed sepsis and septic-shock patients in the emergency department

**DOI:** 10.1017/ash.2022.341

**Published:** 2022-12-06

**Authors:** Esther Y. Bae, Tiffeny T. Smith, Marguerite L. Monogue

**Affiliations:** 1 Department of Pharmacy, The University of Texas Southwestern Medical Center, Dallas, Texas

## Abstract

**Objectives:**

Recognition of sepsis frequently occurs in emergency departments. To evaluate the appropriateness of empiric antibiotic use in the setting of suspected sepsis in emergency department, the percentages of bacterial infection and antibiotic-related adverse drug effects were quantified in an emergency department at an academic medical center.

**Methods:**

We retrospectively reviewed electronic medical records of adults who presented to the emergency department between January 2018 and June 2018 with suspected sepsis (defined as having ≥2 systemic inflammatory response syndrome [SIRS] criteria) and received ≥1 dose of intravenous broad-spectrum antibiotic.

**Results:**

In total, 218 patients were included in the final analysis. Moreover, 19.3% of these patients had confirmed bacterial infections; 44.5% had suspected bacterial infections; and 35.9% did not have bacterial infection. Elevated SIRS score (ie, ≥2) and Quick Sequential Organ Failure Assessment (qSOFA) score (ie, ≥2) were not associated with the presence of bacterial infections. We identified 90-day *Clostridioides difficile* infections in 7 patients and drug-resistant organism infections in 6 patients, regardless of the presence of bacterial infections.

**Conclusions:**

A high number of patients received intravenous broad-spectrum antibiotics in the emergency department without confirmed or suspected bacterial infections that were supported by microbiologic cultures, radiographic imaging, or other symptoms of infections. Most patients who were initially admitted to the emergency department with suspected sepsis were discharged home after receiving 1 dose of intravenous antibiotic. Patients who were initially screened using SIRS score and who received broad-spectrum antibiotics in the emergency department were without confirmed or suspected bacterial infection.

Sepsis is a clinical syndrome caused by the body’s inflammatory response to infection.^1^ It is considered a medical emergency as the delay in its management can lead to multiple-organ system failure and even death. The high mortality risk associated with sepsis has led to national and international societies to publish sepsis management guidelines such as the Surviving Sepsis Campaign (SSC) International Guidelines for the Management of Sepsis and Septic Shock.^2^

The current SSC guidelines recommend initiating antimicrobials within 1 hour in adults with possible septic shock or a high likelihood of sepsis (strong, low quality of evidence) and rapidly assessing the likelihood of infectious versus noninfectious causes of illness in patients with possible sepsis without shock. This early antibiotic initiation recommendation is largely derived from the studies that focused on patients with severe sepsis or septic shock requiring intensive care unit (ICU) admission. Furthermore, these studies are limited by wide patient heterogeneity.^3–5^ Also, up to 40% of patients with sepsis diagnosis do not have an infection; however, many still receive empiric broad-spectrum antimicrobials, even those without hemodynamic instability.^6–8^ The SSC guidelines recommend using sepsis screening tools, such as the systemic inflammatory response syndrome criteria (SIRS) to identify sepsis promptly; however, the guidelines acknowledge the subpar specificity of SIRS score or quick Sequential Organ Failure Assessment (qSOFA) score in identifying infection.^2^

The emergency department (ED) at the study institution utilizes SIRS to help identify patients with sepsis. We evaluated the appropriateness of empiric antibiotic use in the setting of suspected sepsis by describing the presence of bacterial infections in the patients who received empiric antibiotics in the ED.

## Materials and methods

### Study design and setting

The case–control study reviewed electronic medical records (EMR) of patients who presented to the ED at William P. Clements Jr. University Hospital (CUH) at The University of Texas at Southwestern (UTSW) Medical Center between January 1, 2018, and June 30, 2018. The protocol in the ED is to initiate empiric intravenous (IV) broad-spectrum antibiotics within 1 hour of recognition of sepsis based on SIRS scores ≥2 or treating physician’s suspicion of sepsis. Code sepsis at the institution was activated when ED providers utilized the ED sepsis order set.

### Inclusion and exclusion criteria

Adult patients (aged ≥18 years) who presented to the ED and received at least 1 dose of IV broad-spectrum antibiotic(s) in the ED were included in the study. The following IV antibiotics were considered broad-spectrum antibiotics because they were part of the study institution’s ED sepsis order set: aztreonam, cefepime, ceftriaxone, ciprofloxacin, levofloxacin, meropenem, piperacillin-tazobactam, and vancomycin. The following patients were excluded from the study: (1) patients not having SIRS scores ≥2 (heart rate >90 beats per minute; respiratory rate >20 breaths per minute; temperature >38°C or <36°C; and leukocytosis, leukopenia, or bandemia [white blood cells >12,000/mm^3^, <4,000/mm^3^, or bandemia ≥10%]); (2) patients who had received nonprophylactic broad-spectrum antibiotics within 72 hours preceding the ED presentation; and (3) immediate transfers from other hospitals.

### Data collection

The study was approved by the institutional review board at the study institution. *International Classification of Disease, Tenth Revision, Clinical Modification* (ICD-10-CM) codes were utilized to identify patients with certain medical history suggestive of prior immunosuppression. Vital signs and laboratory values (eg, white blood cell count) collected during ED stay were extracted to calculate SIRS and qSOFA scores. If more than one vital parameter or laboratory value was extracted, the most abnormal measurement was used for the analysis. Additional data collected included antibiotics (type and duration) administered in the hospital and/or prescribed at discharge; microbiologic cultures collected in the hospital; and radiographic imaging performed in the hospital.

### Definitions

All patients in the final analysis were categorized into one of the following groups: confirmed bacterial infection (confirmed infection), suspected bacterial infection (suspected infection), or absence of infection. The criteria to define confirmed and suspected infections were derived from an article by Limper et al.^9^ To minimize subjectivity and bias, the primary investigator (E.B.) utilized the Centers for Disease Control and Prevention (CDC) National Healthcare Safety Network (NHSN) Surveillance Definitions to adjudicate suspected infections.^10^ The secondary investigator, a board-certified infectious diseases pharmacy specialist (M.M.), further reviewed the primary investigator’s categorization. Additionally, the investigators reviewed the EMR documentations made by the healthcare professionals during the index hospital stay.

### Outcome measures

The primary outcome was the combined percentage of confirmed and suspected infections. Secondary outcomes included percentages of confirmed, suspected, and absence of infections; in-hospital acute kidney injury (AKI); 90-day *Clostridioides difficile* infections (CDI); 90-day drug-resistant organism (DRO) infections; antibiotic days of therapy (actual and intended); and 30-day all-cause mortality.

### Data analysis

Comparison between patients with and without bacterial infections was performed by utilizing the Pearson χ^2^ test for categorical variables and the Student *t* test for continuous variables. The *P* value for statistical significance was set a priori at .05. Power and anticipated sample size to meet power were not predicted as the study was to include all eligible patients within the time frame.

## Results

In total, 358 patients received IV broad-spectrum antibiotics in the ED during the study period and were assessed for eligibility. Among them, 124 patients were subsequently excluded for not having SIRS scores ≥2; 13 patients were transferred from outside hospitals; and 3 patients received nonprophylactic, broad-spectrum antibiotics within 72 hours preceding the ED arrival. After excluding 141 patients, the final analysis included 218 patients.

The percentages of confirmed, suspected, and absence of infections were 42 (19.3%), 97 (44.5%), and 79 (35.9%), respectively. Among patients who received IV broad-spectrum antibiotics in the ED with a SIRS score ≥ 2, 64% of patients had confirmed or suspected infections.

No significant differences in baseline characteristics were noted among groups (Table 1). The median Charlson comorbidity index scores in both groups were 3, correlating to approximately 59% 10-year mortality risk.^11^ Higher SIRS scores (3 and 4) were not significantly associated with the presence of bacterial infection. However, a higher percentage of patients with bacterial infection had SIRS score of 4 (8.6% vs 2.5%; *P* = .08). Also, 5% of patients in the final analysis had elevated qSOFA (ie, ≥2). Percentages of abnormal body temperature, heart rate, and respiratory rate (components within SIRS) were similar between patients with confirmed or suspected bacterial infections and patients without. A higher number of patients with bacterial infection had abnormal white blood cell count although this was not a significant finding. Higher serum lactic-acid levels (collected in 21 patients) were not significantly associated with the presence of bacterial infection.

**Table 1.** Baseline Demographics of Patients Admitted to the Emergency Department With Suspected Sepsis

Variable	Confirmed and Suspected Infections (N=139), No. (%)^a^	Absence of Infections (N=79), No. (%)^a^	*P* Value^b^
**Patient characteristics**			
Age at admission, median, y (range)	50 (19–97)	54 (18–93)	.087
Sex, male	56 (40.3)	19 (24.1)	**.015**
Race, white^c^	62 (44.6)	40 (50.6)	.39
**Prior comorbidities**			
Charlson comorbidity index, median (range) (n = 102)^d^	3 (1–11)	3 (1–12)	.52
Chemotherapy within last 30 days of admission	19 (13.7)	10 (12.7)	.83
Immunosuppressant(s) at home^e^	3 (2.2)	4 (5.1)	.24
Solid organ transplant history	8 (5.8)	4 (5.1)	.83
HSCT history	9 (6.5)	6 (7.6)	.75
HIV	10 (7.2)	11 (13.9)	.11
**Clinical status upon emergency department admission**			
SIRS score			
2	87 (62.6)	55 (69.9)	.30
3	40 (28.8)	22 (27.8)	.88
4	12 (8.6)	2 (2.5)	.08
Quick SOFA score			
2	5 (3.6)	6 (7.6)	.19
3	0	0	
Glasgow coma score (n=200), median (range)	15 (4–15)	15 (13–15)	.25
Temperature (<36°C or >38°C)^f^	36 (25.9)	21 (26.6)	.91
Heart rate >90 bpm^f^	137 (98.6)	79 (100)	
Respiratory rate >20 bpm^f^	56 (40.3)	36 (45.6)	.45
WBC count <4 or >12 (×10^3^ cells/L)^f^	77 (55.4)	25 (31.6)	.39
**Serum lactic acid (n=21)**			
Median, mmol/L (range)	1.6 (0.7–3.2)	1.8 (0.6–3.7)	.61
≥2 mmol/L	6 (4.3)	2 (2.5)	.50
Pressor requirements	17 (12.2)	7 (8.9)	.44
Code sepsis activated^g^	47 (33.8)	16 (20.3)	**.034**
**Site of electronic medical record (EMR)–documented infections**			
Respiratory	32 (23.0)	18 (22.8)	.97
Skin and soft tissue	17 (12.2)	1 (1.3)	**.0047**
Abdominal	12 (8.6)	9 (11.4)	.51
Genitourinary	63 (45.3)	25 (31.6)	**.048**
Other	15 (10.8)	26 (32.9)	**<.001**
**Antibiotic duration of therapy (DOT)**			
**During ED and/or hospital admission**			
Antibiotic DOT, broad-spectrum, median, d (range)	1.0 (0.5–10.5)	1.0 (0.5–7)	.17
**During ED and/or hospital admission and after discharge**			
Antibiotic DOT, broad-spectrum, median, d (range)	1.0 (0.5–16)	1.0 (0.5–14.5)	**<.001**
Antibiotic DOT, median, d (range)	8.0 (0.5–65)	1.0 (0.5–14.5)	**<.001**
**Discharge from emergency department**			
Types			
Discharged home	115 (82.7)	64 (81.0)	.75
General ward admission	20 (14.4)	15 (19.0)	.37
Intensive care unit admission	4 (2.9)	0 (0)	
Discharged on antibiotics	104 (74.8)	35 (44.3)	**<.001**
Discharged on oral broad-spectrum antibiotics^h^	43 (30.9)	10 (12.7)	**.0025**

Note. HSCT, hematopoietic stem-cell transplant; HIV, human immunodeficiency virus; SOFA, Sequential Organ Failure Assessment; SIRS, systemic inflammatory response syndrome; WBC, white blood cell count; DOT, days of therapy.

a Units unless otherwise specified.

b *P* values are based on the Pearson χ^2^ test for categorical variables and on the Student *t* test for continuous variables.

c Race was determined from medical records.

d Charlson comorbidity index predicts the 10-y mortality based on the types and the numbers of comorbidities. Higher number correlates to a higher predicted mortality risk.

e Excluded chemotherapy medications

f If multiple values were available during emergency and/or hospital admission, the most abnormal value recorded was included in the analysis.

g Code sepsis was triggered when the ED provider utilized the ED sepsis order set.

h Oral broad-spectrum antibiotics were oral alternatives to the intravenous broad-spectrum antibiotics considered in the review (ie, ciprofloxacin, levofloxacin).

Code sepsis at the study institution was activated when the ED providers utilized the sepsis order set, and it was activated more frequently in those with bacterial infections (33.8% vs 20.3%; *P* = .034). Code sepsis was activated in 29% of the patients included in the study.

The most common physician documented infection was genitourinary infection (Table 1), and it was more common in patients with confirmed or suspected bacterial infection (45.3% vs 31.6%; *P* = .048). The most common source of microbiologic culture collected was urinary (Table 2). Among 164 urine cultures that were collected, 51 had positive microbiologic findings. Among the positive urine cultures, 12 were collected from patients without symptoms of urinary tract infection. The second most common EMR-documented infection was respiratory tract infection (Table 1). The most common antibiotic administered in the ED was ceftriaxone followed by vancomycin.

**Table 2.** Distribution of Culture Types Collected and Antibiotics Administered in the Emergency Department

Variable	Confirmed and Suspected Infections		Absence of Infections	
	(N = 139) , No. (%)		(N = 79), No. (%)	
Types of cultures	Total	Positive	Total	Positive
Blood	71 (51.1)	2 (1.4)	38 (48.1)	0 (0)
Sputum	5 (3.6)	0 (0)	0 (0)	0 (0)
Urine	102 (73.4)	39 (28.0)	62 (78.5)	12 (15.2)
Cerebrospinal fluid	1 (0.7)	0 (0)	1 (1.3)	0 (0)
Wound	4 (2.9)	2 (1.4)	0 (0)	0 (0)
Body fluid	1 (0.7)	0 (0)	0 (0)	0 (0)
Stool	1 (0.7)	1 (0.7)	0 (0)	0 (0)
Aspirate	1 (0.7)	0 (0)	0 (0)	0 (0)
Abscess	1 (0.7)	1 (0.7)	0 (0)	0 (0)
**Antibiotics administered**				
Ceftriaxone	78 (56.1)		51 (64.6)	
Vancomycin	40 (28.8)		20 (25.3)	
Piperacillin-tazobactam	19 (13.7)		11 (13.9)	
Cefepime	7 (5.0)		7 (8.9)	
Levofloxacin	15 (10.8)		2 (2.5)	
Ciprofloxacin	6 (4.3)		2 (2.5)	
Aztreonam	3 (2.2)		3 (3.8)	
Meropenem	0 (0)		0 (0)	

Total duration of broad-spectrum antibiotic therapy prescribed in the ED or at admission and/or at discharge varied significantly between patients with and without bacterial infection; the median in-hospital duration of broad-spectrum antibiotics was 1 day in both groups (Table 1). Most patients were discharged home from the ED, and 45% of patients without bacterial infections were discharged home on antibiotics.

No AKIs were identified in our analysis (Table 3). However, 3% of patients were readmitted to the hospital due to *Clostridioides difficile* infections, and another 3% of patients were readmitted to the hospital due to infections with drug-resistant organism within 90 days of the index ED presentation.

**Table 3.** Antibiotic-Related Adverse Drug Effects in Patients Admitted to the Emergency Department With Suspected Sepsis

Variable	Confirmed and Suspected Infections (N = 139), No. (%)	Absence of Infections (N = 79), No. (%)
Acute kidney injury^a^	0 (0)	0 (0)
*C. difficile* infection, 90-d	2 (1.4)	5 (6.3)
Drug-resistant organism infection, 90-d	3 (2.2)	3 (3.8)
Meropenem-resistant *P. aeruginosa*	1 (0.72)	0 (0)
Oxacillin-resistant *S. epidermidis*	2 (1.4)	0 (0)
Vancomycin-resistant *E. faecium*	0 (0)	1 (0.72)
Methicillin-resistant *S. aureus*	0 (0)	1 (0.72)
Cefepime-, pip/tazo-resistant *E. cloacae* complex	0 (0)	1 (0.72)
Mortality, 30-d	1 (0.7)	0 (0)

a Increase in serum creatinine (SCr) by ≥0.3 mg/dL (≥26.5 µmol/L) within 48 h or increase in SCr to ≥1.5 times baseline, which is known or presumed to have occurred within the prior 7 days; or urine volume <0.5 mL/kg/h for 6 h.

## Discussion

In this case–control study, more than one-third of the patients who received IV broad-spectrum antibiotics in the ED for suspected sepsis (SIRS score ≥2) did not have confirmed or suspected bacterial infection supported by positive cultures or clinical findings suggestive of bacterial infection. Several studies report similar findings. A retrospective cohort study by Minderhoud et al^12^ demonstrated that 30% of patients who were started on empiric antibiotic therapy in the ED for suspected sepsis did not have confirmed or suspected bacterial infection. In a similar study focused on ICU patients, Klouwenberg et al^6^ found that almost half of the patients admitted with suspicion for sepsis lacked infection or only had “possible” sepsis, questioning the benefit of antibiotic therapy in many presumed sepsis cases.

In our study, approximately one-third of the patients who had a SIRS score ≥2 had neither confirmed nor suspected infection but received broad-spectrum IV antibiotics. Most of the patients exhibited elevated body temperature upon ED arrival. However, fever is not specific to infection and is linked to numerous noninfectious causes such as medications.^13,14^ Heart rate and respiratory rate, which are also components of SIRS, are additionally nonspecific and easily affected by environmental factors. Abnormal WBC count and serum lactic acid ≥2 mmol/L were not significantly associated with the presence of infection either but were present in a higher number of patients with bacterial infection.

The limitation of sepsis screening tools, such as SIRS and qSOFA, is acknowledged by the sepsis guidelines; however, SIRS continues to be part of the definitions of severe sepsis and septic shock in the Centers for Medicare & Medicaid Services (CMS) Severe Sepsis and Septic Shock Early Management Bundle (SEP-1).^1,5,15^ To improve adherence to the CMS core measures, our institution activates code sepsis in patients with SIRS scores ≥2 to promptly initiate sepsis management, including immediate initiation of empiric IV antibiotics. We cannot speak for the specificity of SIRS in identifying sepsis of bacterial infection; however, the SEP-1 stringent bundle combined with its ineffective definition of sepsis may be leading to increased unwarranted use of antibiotics at the study institution.^5^

Balancing the high mortality risk of sepsis with antimicrobial stewardship is challenging. Studies have shown a significant correlation between rapid administration of antibiotics and lower in-hospital mortality in sepsis.^3,4,16^ However, unnecessary and inappropriate antibiotics are strongly associated with antibiotic-related ADEs as well.^17^ In this study, several readmissions due to CDI and DRO infections were noted, although we are unable to attribute the cause of those infections to the prior antibiotic exposure.

Several other areas of improvement were noted pertaining to the utilization of microbiologic cultures. Blood cultures were collected in only half of the patients despite the SSC recommendation to obtain blood cultures as part of its 2019 hour-1 bundle.^2^ Urinary cultures were collected in majority of the patients; however, <10% of the patients reported urinary symptoms upon chart review. High rate of positive urinary cultures likely contributed to increased antibiotic use despite the current recommendation to withhold antibiotic therapy in asymptomatic bacteriuria.^18^

This study had several limitations. It was retrospective in nature, and the assessment of clinical infection by the investigators was based on documentations available in the EMR. Determination of presence or absence of infection was performed by pharmacists who are not formerly trained diagnosticians. Although the goal of the institutional protocol was to trigger code sepsis in all patients meeting SIRS criteria, this was not done in 71% of patients; therefore, it was difficult to assess whether the antibiotics were administered in the setting of suspected sepsis versus infection. Lastly, the study lacked a negative control group to appropriately assess SIRS or qSOFA for its ability to predict sepsis of bacterial infection.

In conclusion, a notably high proportion of patients with suspected sepsis (SIRS score ≥2) received IV broad-spectrum antibiotics in the ED, but these patients were later found to lack bacterial infection. Most of the patients in the ED were discharged home from ED, further raising the question of whether those patients needed to be initiated on IV broad-spectrum antibiotics. Despite having no bacterial infections, duration of broad-spectrum antibiotics in the ED and/or hospital admission in these patients did not significantly vary from the patients with confirmed or suspected bacterial infection. Lastly, future hospital readmissions due to antibiotic-related ADE were identified in several patients. The combined findings indicate that more stringent and careful evaluation of individual patient is possible and necessary before making the decision to initiate IV antibiotics in the ED.
